# Classifying smoking status using linear and non-linear models based on clinical health records

**DOI:** 10.1007/s00216-026-06584-1

**Published:** 2026-06-10

**Authors:** Murilo de Oliveira Souza, Paulo Roberto Filgueiras, Ian Wilson, Royston Goodacre

**Affiliations:** 1 Laboratory of Analytics, Metabolomics, and Chemometrics, Federal Institute of Espírito Santo, ES-482 Cachoeiro-Alegre, Km 72 - Rive, Alegre, ES 29500-000 Brazil; 2 Centre for Metabolomics Research, Department of Biochemistry, Cell and Systems Biology, Institute of Molecular, Systems and Integrative Biology, University of Liverpool, Crown Street, Liverpool, L69 7ZB UK; 3 Laboratoy of Chemometrics, Federal University of Espírito Santo, Av. Fernando Ferrari, 514 – Goiabeiras, Vitória, ES 29075-910 Brazil; 4 Department of Metabolism, Digestion and Reproduction, Faculty of Medicine, Imperial College London, Du Cane Road, London, W12 0NN UK

**Keywords:** Clinical chemistry, SVM, PLS-DA, Random forest, Logistic regression

## Abstract

Routine clinical chemistry data are widely collected in medical settings, yet their potential for lifestyle characterization using multivariate analysis remains underexplored. Leveraging these routinely available measurements could provide a cost-effective strategy for identifying lifestyle-related biochemical patterns at the population level. In this study, a range of linear and non-linear multivariate classification methods were evaluated to discriminate between smokers and non-smokers using 23 routine clinical chemistry measurements. Linear approaches included traditional partial least squares discriminant analysis (PLS-DA), PLS-DA with bootstrap resampling, and logistic regression (LR), while non-linear models comprised support vector machines (SVM) and random forest (RF). The results revealed differences between linear and non-linear classification strategies. Random forest showed comparatively better classification performance for the present dataset under the evaluated conditions, suggesting its ability to capture complex relationships within the biological data. Variable importance analysis highlighted the cholesterol ratio, total protein, potassium, and lactate dehydrogenase as relevant contributors to class discrimination, suggesting systemic metabolic and physiological differences between smokers and non-smokers. Overall, the findings demonstrate that routinely available clinical chemistry parameters, when coupled with appropriate multivariate analysis, can effectively capture smoking-related biochemical alterations. This study contributes to the fields of clinical chemometrics and data-driven healthcare by demonstrating that standard laboratory measurements can support lifestyle stratification, offering a practical and accessible complementary screening strategy prior to more detailed metabolomic investigations.

## Introduction

“HUSERMET” (HUman SERum METabolome) represented one of the earliest large-scale explorations of metabolic phenotyping (metabolomics/metabonomics) as a means of characterizing population metabotypes. Initiated in 2005, the HUSERMET project collected blood serum samples from approximately 4000 nominally healthy individuals (i.e., with no known disease at the time of sampling), aged between 19 and 81 years, from the Stockport area of Greater Manchester (UK) [1].

For metabolomic analyses, a subset of 1200 serum samples were profiled using gas chromatography–mass spectrometry (GC–MS) and ultra-performance liquid chromatography–mass spectrometry (UHPLC–MS), alongside the concomitant collection of routine clinical chemistry data. These MS-based metabolomics data, following the application of multivariate analysis (MVA) models—such as support vector machines (SVM), random forest (RF), and partial least squares–discriminant analysis (PLS-DA)—enabled the prediction of several demographic traits, including sex, age, and body mass index (BMI), with accuracies ranging from 87 to 92%. Furthermore, specific traits, such as age, were associated with distinct metabolic changes, for example increasing serum concentrations of citrate [1].

In addition, associations were observed between several metabolite markers and standard clinical chemistry measurements. Building on these observations, we very recently revisited the demographic metadata collected from HUSERMET participants. Specifically, we applied the same algorithms previously used for metabolomic data analysis (i.e., SVM, RF, and PLS-DA) to the clinical chemistry data alone and demonstrated that samples could also be accurately characterized according to age, BMI, and sex [2]. Notably, these results were highly consistent with those obtained using metabolomic profiling [1]. Moreover, the integration of metabolomics with clinical chemistry data further improved classification performance, enabling more accurate quantitative predictions of age and BMI. Collectively, these findings reflect a growing interest in the use of clinical chemistry data combined with multivariate analysis for biomarker discovery, adding value to their application in areas such as disease stratification [2–4].

Considering these observations, and given the wide availability of clinical chemistry data, we sought to investigate whether similar approaches could be extended to other domains, such as lifestyle characterization. One particularly relevant lifestyle factor is smoking and, in the original HUSERMET study, we had noted a wide range of metabolic changes that correlated with smoking including the amino acid tryptophan (previously associated with smoking initiation and nicotine exposure [5, 6]). As smoking is widely recognized as a major risk factor for lung cancer and cardiovascular disease, we wished to see if routinely collected clinical data could be used to identify smoking-related physiological effects in individuals undergoing routine health screening (as well as for identifying early changes associated with disease onset and progression [7]). We therefore took the opportunity to employ a range of multivariate analysis (MVA) techniques to examine the potential of using the “classical” clinical chemistry measurements obtained in the HUSERMET study to identify smokers as a model lifestyle factor. By comparing qualitatively different classification strategies applied to a smoker versus non-smoker dataset based on 23 clinical chemistry measurements, we have investigated their potential to capture lifestyle-related biochemical signatures.

## Materials and methods

### Study background and ethical compliance

For HUSERMET, serum samples and clinical data were originally collected by Dunn et al. [1] which focused on metabolic profiling using MS-based methods and explored correlations between clinical chemistry markers and metabolite levels. The clinical data were subsequently explored by Xu et al. [2] for patient phenotyping (age, sex, BMI). The study was approved by the Stockport Research Ethics Committee, and all participants provided written informed consent in accordance with the WMA Declaration of Helsinki and the NIH Belmont Report.

### Dataset preparation

Initially, the clinical chemistry data from 1200 individuals were considered. Variables and samples with ≥ 20% missing data were excluded, and any remaining gaps were imputed using the median of each variable due to its robustness to outliers. After preprocessing, 406 samples remained: 94 self-reported smokers and 312 non-smokers. To balance the dataset, 94 non-smokers were selected using the Kennard–Stone (KS) algorithm. This resulted in a working dataset of 188 samples (94 smokers and 94 non-smokers). Thus, the final dataset contained an equal number of samples in each class, avoiding class imbalance.

For each sample there were 23 clinical chemistry measurements and these included the following: subject age (1), body mass index (2), systolic blood pressure (3), diastolic blood pressure (4), total protein (5), creatinine (6), serum glucose (7), serum sodium (8), potassium (9), serum calcium (10), total cholesterol (11), triglycerides (12), high-density lipoprotein cholesterol, HDL (13), low-density lipoprotein cholesterol, LDL (14), cholesterol ratio, defined as total cholesterol to HDL cholesterol ratio (15), blood urea nitrogen (16), total bilirubin (17), alkaline phosphatase, ALP (18), alanine aminotransferase, ALT (19), aspartate aminotransferase, AST (20), gamma-glutamyl transferase, GGT (21), phosphate (22), and lactate dehydrogenase, LDH (23).

Binary classification models (PLS-DA, LR, SVM, and RF) were randomly divided using a stratified random split into training (70%; 66 smokers and 66 non-smokers) and test (30%; 28 smokers and 28 non-smokers) sets, preserving class proportions in both subsets. Subsequently, autoscaling was applied to all models except RF, which was built using raw data [8–10]. All analyses were conducted in MATLAB® R2018a (The MathWorks, Natick, MA, USA) using in-house scripts and external toolboxes.

### Partial least squares-discriminant analysis (PLS-DA) methods

The optimal number of latent variables (LVs) in the PLS-DA model was determined through five-fold Venetian blinds cross-validation, selecting the solution that minimized the cross-classification error. Model construction was carried out using the Classification Toolbox (Milano Chemometrics and QSAR Research Group, version 6.0, 2013) [11], in combination with in-house scripts.

A PLS-DA bootstrap resampling approach with replacement was applied to the full dataset (188 samples) to generate 1000 PLS-DA models. Each model was trained on a resampled subset, and its performance was evaluated using the corresponding out-of-bag (OOB) test samples. A permutation test (1000 iterations) was conducted by scrambling class labels to assess statistical significance [12–14]. This approach enabled comparison of the observed model performance against the null distribution to assess statistical significance and rule out classification due to random chance. Data were autoscaled prior to modeling. All procedures were carried out using the Cluster Toolbox (https://github.com/Biospec/), in combination with in-house scripts [15].

### Logistic regression (LR)

A LR model was constructed using the same sets used in PLS-DA. LR estimates the probability of a sample belonging to a given class (e.g., smoker) by applying a logistic (sigmoid) function to a linear combination of predictor variables. This transforms the output into a bounded probability between 0 and 1, making it suitable for binary classification problems. Model parameters are estimated by maximum likelihood, and the resulting coefficients can be interpreted in terms of odds ratios, which quantify the effect of each variable on the outcome likelihood [16]. The LR model was built using the fitglm function from the Statistics Toolbox package in Matlab®. All variables were used as initial input. After building the model, the most significant variables (*α* less than 10%) were selected for the final model. This final model was built with only 11 variables out of the initial 23, namely, subject age (1), body mass index (2), total protein (5), serum sodium (8), potassium (9), serum calcium (10), total cholesterol (11), triglycerides (12), high-density lipoprotein cholesterol, HDL (13), total CHOL/HDLC (15), and lactate dehydrogenase, LDH (23).

### Random forest (RF)

RF is an ensemble learning method in which each base learner is a decision tree, constructed by combining classification and regression trees (CART) with bootstrap aggregating (bagging) [17]. Tree growth was based on the Gini impurity criterion used for node splitting, whereby node splits were selected to maximize the reduction in class impurity. No data preprocessing was applied as RF is robust to differences in scale and distribution across variables. During training, each tree was built using a bootstrap sample (in-bag set) comprising approximately two-thirds of the training subset (from the ~ 70% of samples selected by the stratified random split, as described previously) [9, 18]. The remaining one-third, known as the out-of-bag (OOB) set, was used for internal validation and performance estimation. The remaining 30% of samples constituted the external test set.

Hyperparameter optimization was performed through an iterative evaluation of different RF configurations, aiming to reduce overfitting and improve external generalization performance. The number of trees (100, 400, and 1000), minimum leaf size (50, 100, and 150), and mtry (number of variables tried at each node) percentage (30% and 60% of the original variables) were systematically evaluated. The final model was not selected solely based on the highest training accuracy, but rather on the balance between predictive performance and model robustness, as indicated by OOB and external test results. Final model selection prioritized configurations showing reduced discrepancy between internal and external validation performances. The final RF configuration consisted of 400 trees, minimum leaf size of 100, and mtry corresponding to 30% of the original variables.

Variable importance was not derived from Gini decrease or permutation importance; instead, it was assessed based on the frequency with which each predictor was selected during the construction of the decision trees across the ensemble. Absolute and relative selection frequencies were calculated, and variables exceeding a predefined threshold (25% of the maximum observed selection frequency) were considered relevant contributors to the classification model. This frequency-based strategy was adopted to provide a more stable interpretation of variable relevance across the ensemble, rather than relying solely on impurity-based metrics.

Class probabilities were computed for training (In-Bag and OOB) and external test sets. Additionally, OOB predictions were considered an important indicator of model generalization, as they provide an internal validation estimate using samples not included in the bootstrap construction of individual trees. All RF models were developed and evaluated using in-house MATLAB scripts (available via https://github.com/Biospec/).

### Support vector machine (SVM)

The SVM with a radial basis function (RBF) kernel was employed. The RBF kernel, suitable for modeling non-linear patterns, was therefore used to model the data. In this context, the cost parameter (*C*) controls the trade-off between margin maximization and classification error, while the kernel parameter (*γ*) influences the flexibility of the decision boundary [19, 20]. The SVM model was built using the fitglm and fitcecoc functions from the Statistics and Machine Learning Toolbox package. A grid-search strategy was used to evaluate different combinations of *C* and *γ*, with *C* values explored in the range of 10^–3^–10^4^ and *γ* values in the range of 10^–6^–10^4^ (applying logarithmic interpolation). Model performance during parameter selection was assessed using Venetian blinds cross-validation with ten groups, and the optimal hyperparameters (*C* = 666 and *γ* = 0.0044) were selected based on the minimum cross-validation error. The final model was trained with 77 support vectors.

### Performance parameters

Model performance was assessed using the following metrics: false positive rate (FPR), false negative rate (FNR), sensitivity (SEN), specificity (SPE), accuracy (ACC), precision (PRE), F1-score, Balanced Accuracy (BAC), and Matthews correlation coefficient (MCC), as defined in Eqs. 1–9 [13, 21]:

1 $$\mathrm{F}\mathrm{P}\mathrm{R}=\frac{FP}{FP+TN}\times 100$$

2 $$\mathrm{F}\mathrm{N}\mathrm{R}=\frac{FN}{FN+TP}\times 100$$

3 $$\mathrm{S}\mathrm{E}\mathrm{N} \left(\mathrm{r}\mathrm{e}\mathrm{c}\mathrm{a}\mathrm{l}\mathrm{l}\right)=\frac{TP}{TP+FN}$$

4 $$\mathrm{S}\mathrm{P}\mathrm{E}=\frac{TN}{TN+FP}$$

5 $$\mathrm{A}\mathrm{C}\mathrm{C}=\frac{(TP+TN)}{(FN+TP+FP+ TN)}$$

6 $$\mathrm{P}\mathrm{r}\mathrm{e}\mathrm{c}\mathrm{i}\mathrm{s}\mathrm{i}\mathrm{o}\mathrm{n}=\frac{TP }{TP+FP}$$

7 $$\mathrm{F}1=2 \times \frac{Precision \times Recall }{Precision+ Recall}$$

8 $$\mathrm{B}\mathrm{a}\mathrm{l}\mathrm{a}\mathrm{n}\mathrm{c}\mathrm{e}\mathrm{d} \mathrm{A}\mathrm{c}\mathrm{c}\mathrm{u}\mathrm{r}\mathrm{a}\mathrm{c}\mathrm{y} =\frac{SEN+SPE }{2}$$

9 $$\mathrm{M}\mathrm{C}\mathrm{C}=\frac{TP.TN-FP.FN}{\sqrt{\left(TP+FP\right).\left(TP+FN\right).\left(TN+FP\right).(TN+FN)}}$$

True Positive (*TP*) denotes smoker samples correctly classified as such. At the same time, a False Negative (*FN*) indicates the number of smoker samples incorrectly classified as non-smoker. Conversely, True Negative (*TN*) is the number of non-smoker samples correctly classified as non-smoker, and False Positive (*FP*) represents the non-smoker samples incorrectly classified as smoker. The MCC condensed the confusion matrix into a single value, ranging from −1 (total disagreement) to + 1 (perfect classification), with 0 indicating random prediction [22].

### Box-and-whisker plots

The normality of the variables was assessed separately for smokers and non-smokers using the Shapiro–Wilk test. Variables with *p* > 0.05 were considered normally distributed. For variables showing normality in both groups, an independent-samples *t*-test was applied: Student’s *t*-test was used when variances were equal, and Welch’s *t*-test was applied when variances were significantly different. For variables that did not follow a normal distribution in at least one of the groups, the non-parametric Mann–Whitney *U* test was used to compare medians between groups [23–25]. Values with *p* ≥ 0.05 were considered not significantly different between smokers and non-smokers, whereas values with *p* < 0.05 were considered significantly different.

## Results and discussion

The overall performance of the models evaluated to differentiate between smoker and non-smoker samples using three linear methods (traditional PLS-DA, PLS-DA with bootstrap resampling, and logistic regression) and two non-linear methods (Random Forest and SVM) is summarized in Table 1. The aim of this study was to compare qualitatively different classification approaches applied to a smoker versus non-smoker dataset based on 23 clinical chemistry measurements, rather than to establish a statistically definitive best-performing model.

**Table 1:** Performance parameters obtained by linear and non-linear methods on the test sets only. Values are reported on a 0–1 scale and expressed as percentages when discussed in the text

Model	PLS-DA	PLS-DA bootstrap	Logistic regression (LR)	Random forest (RF)	SVM
	*(n* = *28 smokers and 28 non-smokers)*	*n* = *188 samples*	*(n* = *28 smokers and 28 non-smokers)*	*(n* = *28 smokers and 28 non-smokers)*	*(n* = *28 smokers and 28 non-smokers)*
SEN	0.750	0.699	0.607	0.857	0.750
SPE	0.536	0.688	0.714	0.750	0.786
ACC	0.643	–	0.661	0.804	0.768
PRE	0.618	–	0.680	0.774	0.778
F1-score	0.677	–	0.642	0.814	0.764
BAC	0.643	–	0.661	0.804	0.768
MCC	0.293	–	0.323	0.612	0.536
CCR	–	0.693	–	–	–

Autoscaling was applied to all models, except for RF. For PLS-DA, LR, SVM, and RF, training comprised 70% of each class (*n* = 66 smokers and *n* = 66 non-smokers), and the external test set included the remaining 30% of each class (*n* = 28 smokers and *n* = 28 non-smokers), using a stratified random split strategy. RF model was built with 400 trees, minimum leaf size of 100, and mtry corresponding to 30% of the original variables. RF metrics reported in the table correspond to the external test performance. PLS-DA values represent averages from 1000 bootstrap resampling iterations based on test set predictions of the y-variable

*SEN *sensitivity, *SPE *specificity, *ACC *accuracy, *PRE *precision, *BAC *Balanced Accuracy, *MCC *Matthews correlation coefficient, *CCR *average correct classification rate

These models showed that non-linear classifiers outperformed linear approaches; however, the interpretation of this superiority should be considered with caution, since model performance may also be influenced by optimization strategy, validation methodology, and dataset partitioning. Nevertheless, RF and SVM demonstrated comparatively better predictive performance under the evaluated conditions, likely due to their ability to better capture complex and non-linear relationships between variables. Moreover, this finding aligns with previous studies reporting the higher efficiency of non-linear models when dealing with biological data and complex patterns, as observed in this experiment involving clinical parameters derived from biological fluids [1, 26, 27]. The precision (PRE), F1-score, and Balanced Accuracy (BAC) highlight differences in model performance. Non-linear models (RF and SVM) showed comparatively higher PRE (0.774 and 0.778), F1-score (0.814 and 0.764), BAC (0.804 and 0.768), and MCC (0.612 and 0.536), respectively, suggesting a more balanced prediction across both classes under the evaluated conditions. In contrast, PLS-DA presented lower PRE (0.618), F1-score (0.677), BAC (0.643), and MCC (0.293), indicating a more limited predictive performance and a less balanced sensitivity/specificity profile for the present dataset.

### Linear methods

PLS-DA exhibited moderate predictive performance, with 75.0% sensitivity, 53.6% specificity, and 64.3% accuracy on the test set. The low specificity indicates a tendency toward false positives; i.e., non-smoker samples misclassified as smokers. Training set results (78.8% sensitivity, 83.3% specificity, and 81.1% accuracy; not shown in Table 1) suggest some degree of overfitting and limited model generalization. In particular, the substantial reduction in specificity from the training to the external test set indicates reduced robustness in the discrimination of non-smoker samples. Nevertheless, this configuration represented the best-performing PLS-DA model obtained for the present dataset, using 7 latent variables (LVs) and yielding a cross-validation error rate of 0.17. Overall, these findings indicate that PLS-DA had limited ability to robustly discriminate between smoker and non-smoker samples under the evaluated conditions.

**Fig. 1:**
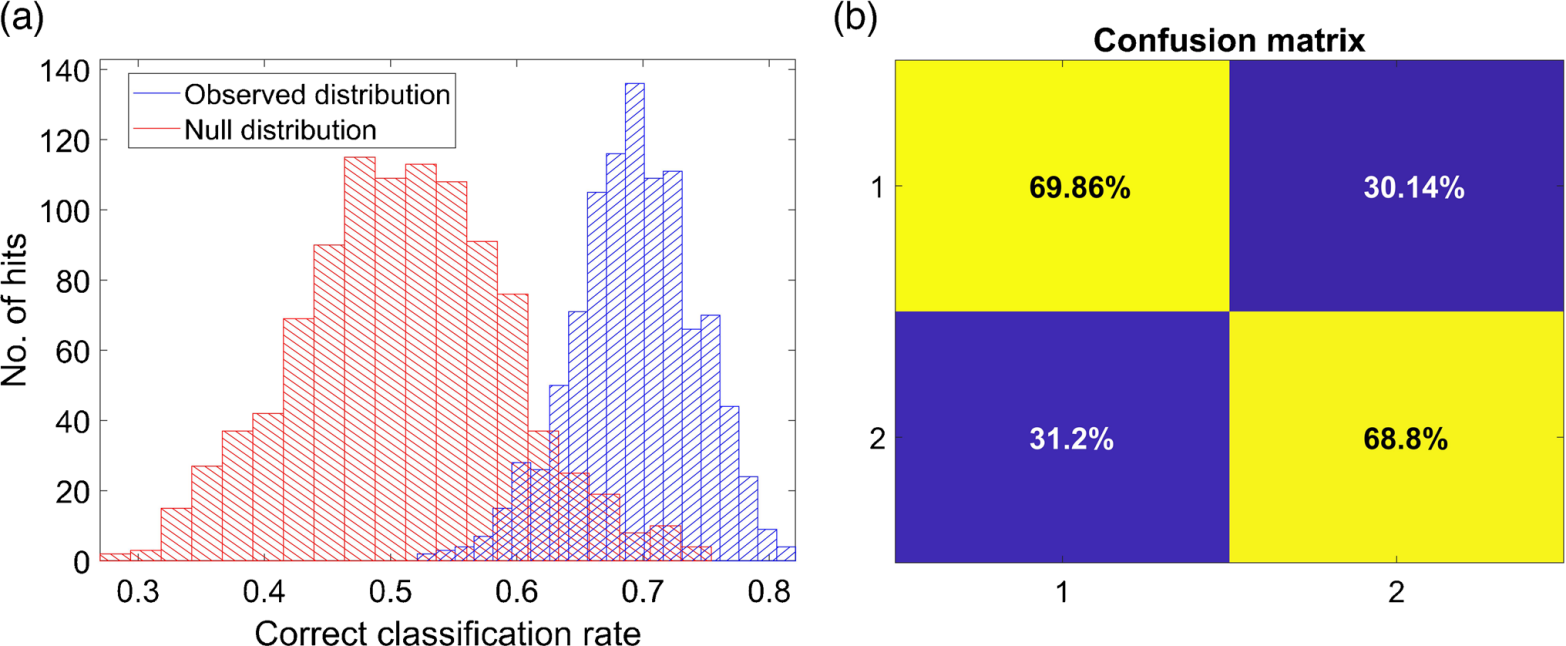
**a** Results are based on 1000 bootstrap iterations with replacement, where only the test set results are shown. Blue histograms represent the observed distributions of correct classification rates from the real test sets, while red histograms show the null distributions from permutation testing and **b** average performance metrics from PLS-DA based on 1000 bootstrap resampling iterations. Classification and misclassification rates for class 1 (smoker) and class 2 (non-smoker) were derived from the mean average confusion matrix

To enhance model evaluation, PLS-DA with bootstrap resampling was applied to the entire dataset (*n* = 188) without splitting into separate training and test sets. Due to the resampling with replacement strategy, on average, each model used 63.2% of the data in the training set and 36.8% in the test set, and any plots are shown from the test set predictions only [28]. The CCR distributions from 1000 test sets are shown in Fig. 1a, where observed values are provided in blue and null distributions from permutation testing in red [13, 29]. In Fig. 1b, classification and misclassification rates for class 1 (smoker) and class 2 (non-smoker), based on the confusion matrix from the mean average of these 1000 test sets, are summarized.

Model performance is illustrated in Fig. 1a by comparing the observed CCR distribution to a random (null) reference. A rightward shift of the observed distribution indicates enhanced classification capability. Both distributions were generated through bootstrap validation of the PLS-DA models, resulting in a correct classification rate of 69.3% (*p*-value = 0.030) under autoscaling. As shown in Fig. 1b, class-specific prediction accuracy was moderate, with sensitivity of 69.9% for smokers and specificity of 68.8% for non-smokers.

The binary logistic regression model demonstrated moderate performance on the test set (Table 1), with sensitivity, specificity, and overall accuracy values of 60.7%, 71.4%, and 66.1%, respectively. These results indicate only moderate discrimination between smokers and non-smokers, particularly due to the relatively lower sensitivity. In the training set (results not shown in Table 1), the model achieved 78.8% for sensitivity, specificity, and accuracy, consistent with the performance of a moderately effective classification model. However, the better performance observed in the training set compared to the test set suggests that some degree of overfitting cannot be excluded. Compared to PLS-DA, logistic regression showed similar overall accuracy, with slightly better specificity but slightly lower sensitivity in the test set. Overall, these findings suggest limited ability of linear models to capture more complex patterns present in biological data, supporting the application of non-linear classification approaches to improve predictive robustness and classification performance.

### Non-linear methods

The RF model demonstrated comparatively better predictive performance than the evaluated linear methods, particularly during OOB internal validation, which is commonly used to estimate model generalization in RF [10], Table 2.

**Table 2:** Performance parameters obtained by random forest method (Ntree = 400)

Parameters	Training subset (in-bag set) *(n* = *66 smokers and 66 non-smokers)*	Test set	
		Internal validation (out-of-bag (OOB)) *(n* = *66 smokers and 66 non-smokers)*	External test set *(n* = *28 smokers and 28 non-smokers)*
SEN	1.000	0.864	0.857
SPE	1.000	0.848	0.750
ACC	1.000	0.856	0.804
MCC	1.000	0.712	0.612

Values are reported on a 0–1 scale and expressed as percentages when discussed in the text

*SEN *sensitivity, *SPE *specificity, *ACC *accuracy, *MCC *Matthews correlation coefficient

**Fig. 2:**
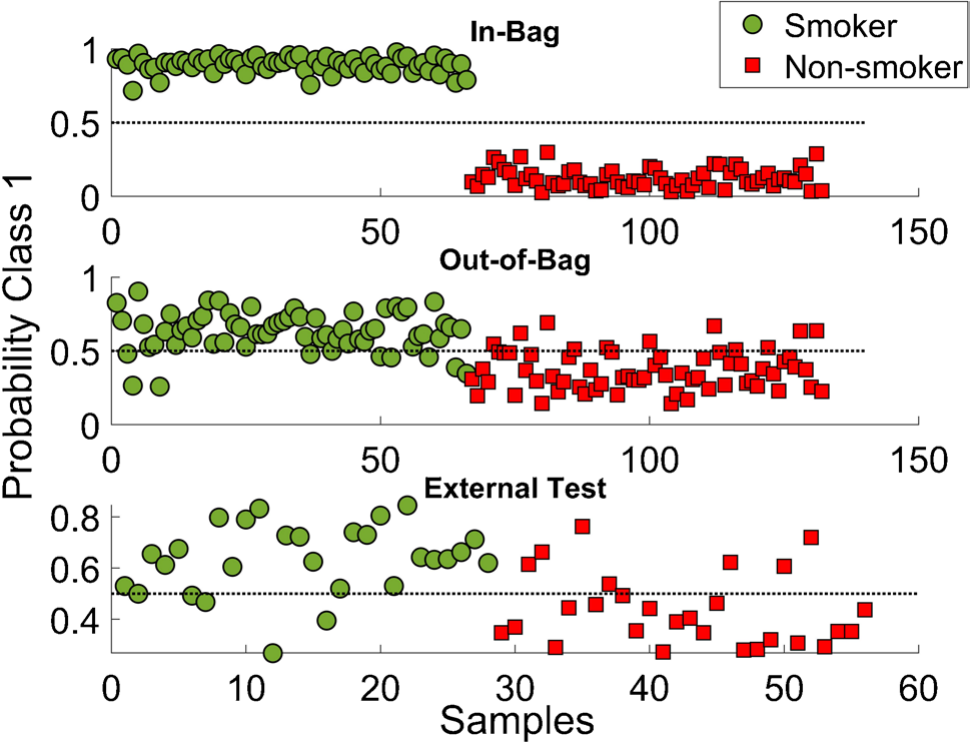
Classification probabilities for the random forest model across training (in-bag), internal validation (out-of-bag), and external test sets. Each point represents a sample predicted as *smoker* (green circles) or *non-smoker* (red squares). The dotted line (0.5) denotes the decision threshold for class assignment

The RF model exhibited comparatively higher performance during OOB validation (ACC = 85.6%, MCC = 0.712), with relatively balanced sensitivity and specificity. On the external test set, performance decreased (ACC = 80.4%, MCC = 0.612), reflecting a more realistic estimate of predictive ability on unseen samples. Importantly, the relatively small discrepancy between OOB and external test performances suggests acceptable model stability and generalization under the evaluated conditions. Since OOB samples are not used during the construction of individual trees, comparison between OOB and external test results provides a useful assessment of model generalization. Although some reduction in predictive performance between internal and external validation remained, this behavior is expected in relatively small clinical datasets and likely represents a more realistic estimation of external predictive performance. Overall, the RF model maintained predictive metrics above random expectation, suggesting the presence of biologically relevant discriminatory information within the evaluated clinical variables (Fig. 2).

**Fig. 3:**
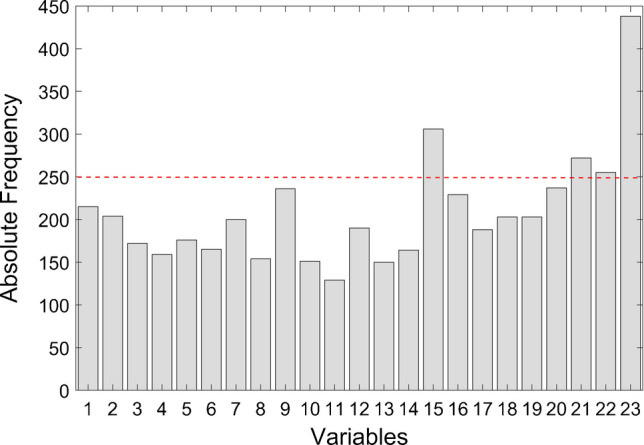
Relative frequency plot of RF model. Variables numbered from 1 to 23 represent the following: subject age (1), body mass index (2), systolic blood pressure (3), diastolic blood pressure (4), total protein (5), creatinine (6), serum glucose (7), serum sodium (8), potassium (9), serum calcium (10), total cholesterol (11), triglycerides (12), high-density lipoprotein cholesterol (HDL) (13), low-density lipoprotein cholesterol (LDL) (14), cholesterol ratio, total CHOL/HDLC (15), blood urea nitrogen (16), total bilirubin (17), alkaline phosphatase (ALP) (18), alanine aminotransferase (ALT) (19), aspartate aminotransferase (AST) (20), gamma-glutamyl transferase (GGT) (21), phosphate (22), and lactate dehydrogenase (LDH) (23). Note: All variables exceeded the minimum frequency threshold adopted in the RF model (25% of the maximum observed selection frequency). The red dashed line was added only as a visual reference to emphasize variables selected more than 250 times during RF model construction. This value does not represent a statistical significance threshold

Variable frequency analysis (Fig. 3) showed that all 23 clinical variables exceeded the minimum relevance threshold adopted in this study, suggesting that all parameters contributed to RF model construction to some extent. Nevertheless, total CHOL/HDLC ratio (15), gamma-glutamyl transferase (GGT, 21), phosphate (22), and lactate dehydrogenase (LDH, 23) exhibited comparatively higher selection frequencies across the ensemble.

CHOL/HDLC ratio (15), GGT (21), and LDH (23) are biologically consistent with physiological and metabolic alterations reported in the literature. Smoking has been associated with dyslipidemia and metabolic dysregulation, including increased cholesterol, LDL (14), and triglyceride levels (12), as well as reduced HDL (13) concentrations, contributing to cardiovascular risk and insulin resistance [5]. Smoking exposure has also been linked to oxidative stress and chronic inflammation, reflected by alterations in metabolites involved in redox balance and antioxidant pathways [5, 30]. In addition, elevated liver-related enzymes, including ALP (18), ALT (19), and AST (20), have been associated with hepatocellular dysfunction and systemic metabolic disturbances in smokers [5]. LDH is a well-established biomarker of cellular and tissue injury, since it is released into circulation following membrane damage or altered cellular metabolism, and increased serum LDH activities have been reported in several human diseases associated with systemic dysfunction and inflammation [31].

**Fig. 4:**
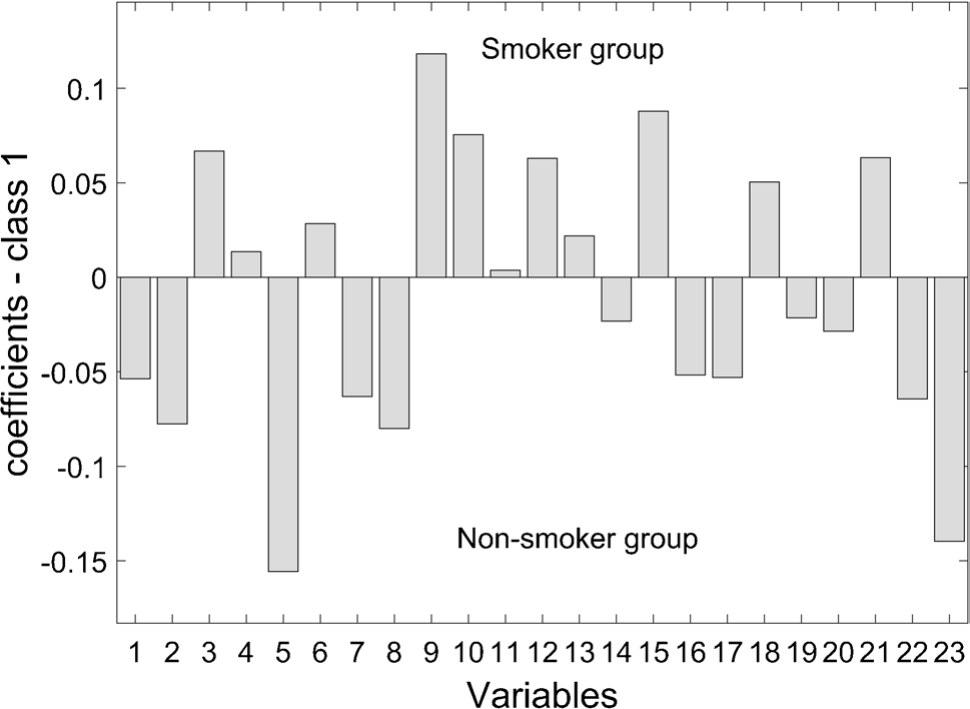
Regression coefficients calculated from the PLS-DA model to identify variables that contribute most strongly to class discrimination. Variables numbered from 1 to 23 represent the following: subject age (1), body mass index (2), systolic blood pressure (3), diastolic blood pressure (4), total protein (5), creatinine (6), serum glucose (7), serum sodium (8), potassium (9), serum calcium (10), total cholesterol (11), triglycerides (12), high-density lipoprotein cholesterol (HDL) (13), low-density lipoprotein cholesterol (LDL) (14), cholesterol ratio (CHOL/HDLC) (15), blood urea nitrogen (16), total bilirubin (17), alkaline phosphatase (ALP) (18), alanine aminotransferase (ALT) (19), aspartate aminotransferase (AST) (20), gamma-glutamyl transferase (GGT) (21), phosphate (22), and lactate dehydrogenase (LDH) (23). Positive coefficients indicate association with smokers; negative coefficients favor non-smokers

Although RF effectively identifies influential variables, its ensemble nature limits direct interpretation of the direction and magnitude of each variable’s contribution to class prediction. Therefore, complementary analyses such as PLS-DA regression coefficients (see Fig. 4) were employed to explore the biological meaning of these discriminating markers.

As an alternative non-linear classifier, we used SVM with an RBF kernel. The SVM model showed high training performance (1.000 for all evaluated parameters; results not shown in Table 1) and reduced predictive performance on the external test set (see Table 1). However, the relatively large number of support vectors (~58% of the 132 samples used in the training set) and the decrease in performance between training and test sets suggest a complex decision boundary, indicating that some degree of overfitting cannot be excluded. In SVM models, support vectors define the decision boundary, and a relatively high number of support vectors may reflect the intrinsic complexity and partial overlap commonly observed in biological data, which may require more flexible non-linear separation boundaries.

### Identification of predictive clinical markers

The RF and SVM models, capable of capturing non-linear relationships, showed comparatively better predictive performance under the evaluated conditions. However, these methods provide limited biological interpretability: SVM operates largely as a “black box,” and RF indicates variable usage frequency but not their specific impact on class separation. In contrast, although the PLS-DA model showed only moderate discriminative performance, it remained useful as an exploratory and interpretative chemometric tool. Specifically, the PLS-DA regression coefficients allowed identification of the variables most associated with smokers and non-smokers and provided information regarding the directionality of variable contributions between classes (Fig. 4).

Variables associated with cardiovascular and metabolic status, including systolic blood pressure (3), triglycerides (12), and the CHOL/HDLC ratio (15), showed positive coefficients toward the smoker group, in agreement with previous studies linking smoking to hypertension, dyslipidemia, and metabolic disturbances [5, 30–32]. Liver-related markers, particularly ALP (18) and GGT (21), also exhibited positive coefficients in smokers, supporting previous reports describing smoking-associated oxidative stress, systemic inflammation, and hepatocellular dysfunction. The large-scale HUSERMET study also observed similar variations in ALP, AST, and other markers among healthy individuals, with smoking influencing serum metabolite patterns [1]. In addition, potassium (9) and calcium (10) showed positive coefficients toward smokers, suggesting possible systemic physiological and metabolic alterations associated with smoking exposure [33]. Conversely, non-smokers (negative coefficients) exhibited contributions of total protein (5) and bilirubin (17), indicating a comparatively more favorable biochemical profile. This observation is consistent with studies reporting reduced bilirubin and protein levels, as well as increased liver enzyme activities, in smokers [34, 35]. Bilirubin, an endogenous antioxidant, may protect against oxidative stress in non-smokers [35]. Interestingly, LDH (23) showed a negative coefficient, indicating greater association with the non-smoker group in the PLS-DA model. Although LDH is widely recognized as a non-specific biomarker of tissue injury, systemic dysfunction, inflammation, and altered cellular metabolism, its specific association with smoking exposure remains inconsistent in the literature [31]. It is also important to consider that all individuals included in the present study were clinically healthy at the time of sample collection, including smokers, which may partially explain the absence of elevated LDH contributions toward the smoker group. Therefore, the contribution of LDH observed in this study should be interpreted cautiously and may reflect dataset-specific multivariate relationships rather than a direct smoking-related biological effect.

**Fig. 5:**
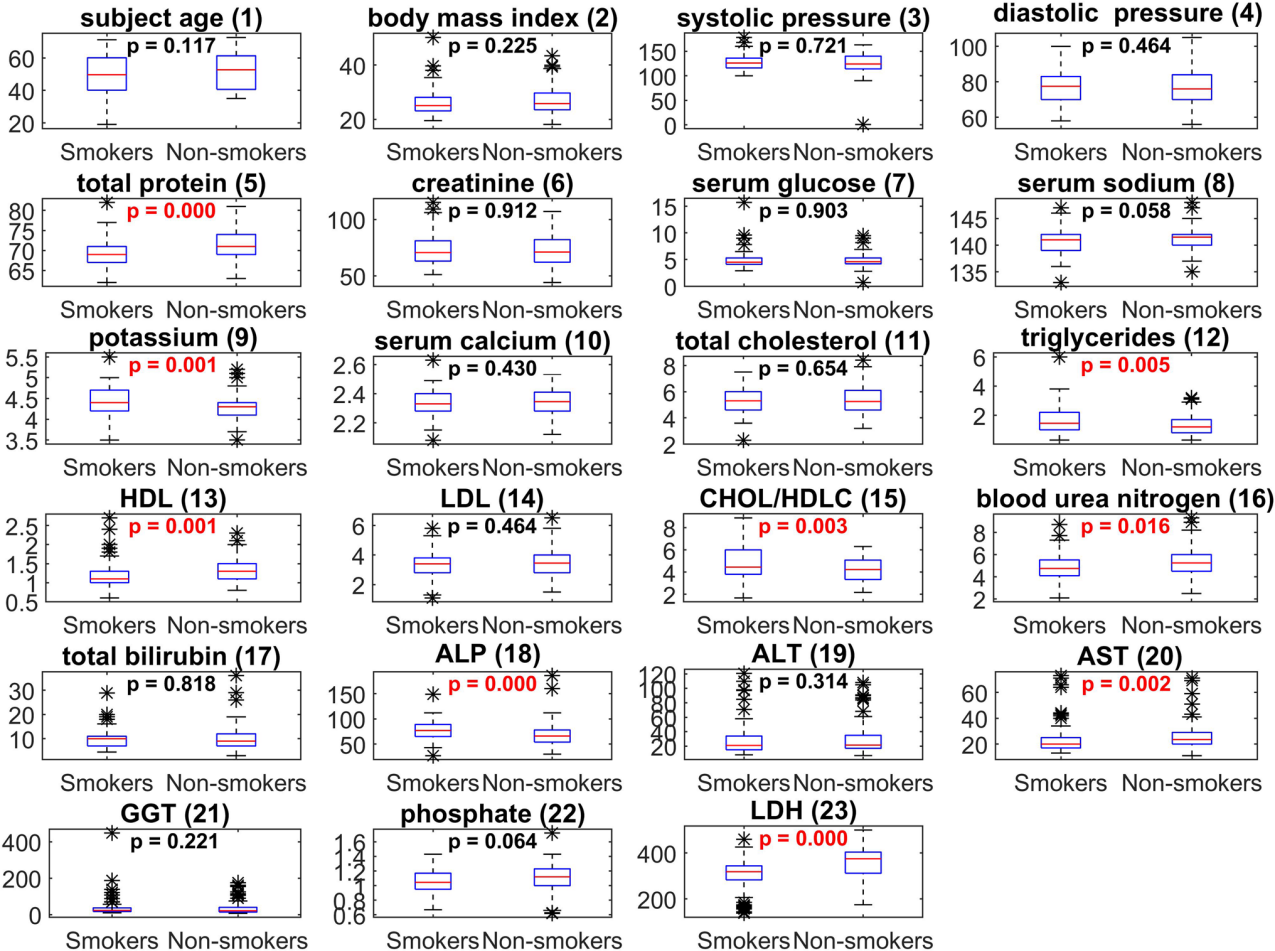
Comparison of clinical and biochemical variables between smokers and non-smokers. Each boxplot represents the data distribution in each group, with the central line indicating the median, the box representing the interquartile range (25–75%), and the whiskers showing the minimum and maximum values, excluding outliers, which are shown as asterisks. Data normality was assessed using the Shapiro–Wilk test; for normally distributed variables, the Student’s *t*-test was applied, and for non-normal variables, the Mann–Whitney test was used. *p*-values < 0.05 indicate statistically significant differences and are highlighted in red

Boxplots of all 23 variables were generated (Fig. 5), with normality assessed by Shapiro–Wilk test. Student’s *t*-test or Mann–Whitney test was applied as appropriate.

Significant differences between smokers and non-smokers were observed for total protein (5) (*p* = 0.0005, Mann–Whitney), potassium (9) (*p* = 0.0009, Mann–Whitney), triglycerides (12) (*p* = 0.0046, Mann–Whitney), HDL cholesterol (13) (*p* = 0.0009, Mann–Whitney), cholesterol ratio (CHOL/HDLC, 15) (*p* = 0.0030, *t*-test), blood urea nitrogen (16) (*p* = 0.0157, Mann–Whitney), alanine aminotransferase (ALT, 19) (*p* = 0.0003, Mann–Whitney), aspartate aminotransferase (AST, 20) (*p* = 0.0022, Mann–Whitney), and lactate dehydrogenase (LDH, 23) (*p* = 7.84 × 10^−8^, Mann–Whitney). All other variables did not show statistically significant differences (*p* ≥ 0.05) (Fig. 5).

Although boxplots accounted for data non-normality, they provide only univariate information. In contrast, the regression coefficients from the PLS-DA model (multivariate) allowed identification of the variables with the greatest influence on class discrimination. The variables most frequently selected in the RF model (Fig. 3), cholesterol ratio (15), and LDH (23), also showed statistical differences in the boxplot distributions between smokers and non-smokers. Similarly, variables with the highest regression coefficients in the PLS-DA model (Fig. 4), total protein (5), potassium (9), and LDH (23), also exhibited statistical differences between the two groups in the boxplot analysis. The lower contribution of some variables in the PLS-DA and RF models, despite significant univariate differences observed in the boxplot analysis (*p* < 0.05), may be associated with covariance, redundancy, and multicollinearity effects among correlated clinical variables within the multivariate structure of the models. For example, liver-related markers such as ALP (18) and AST (20) showed significant individual differences between groups but comparatively lower contributions in the multivariate analyses. Given that most variables did not follow a normal distribution, non-parametric methods such as SVM and RF may provide improved modeling performance, consistent with the results observed in this study. These findings highlight the importance of explainability tools for interpreting complex models and encourage further research to improve the transparency of black-box algorithms such as SVM.

## Conclusions

This study provides a systematic chemometric comparison of linear and non-linear classification methods for discriminating smokers and non-smokers using routine clinical chemistry data. The results indicated that linear classifiers, including PLS-DA and logistic regression, showed limited discriminative performance for the present dataset and under the evaluated conditions. In contrast, random forest, a non-linear method, showed comparatively better classification performance for the present dataset under the evaluated conditions, suggesting its ability to capture complex relationships present in these biological data. Although the SVM model did not substantially outperform the linear approaches, its performance and complex decision boundary also suggested the presence of non-linear relationships within the biological data. In addition, the variables identified by the models, particularly those related to lipid regulation, cardiovascular status, hepatic metabolism, and oxidative stress, were biologically consistent with previous reports describing smoking-associated metabolic and physiological alterations, supporting the interpretability of the observed classifications.

From a chemometric perspective, this work underscores the importance of aligning model complexity with data structure and classification objectives. More broadly, the findings demonstrate that routinely available clinical chemistry measurements, often regarded as low-informative when considered individually, can provide meaningful discriminatory information when analyzed multivariately. This approach offers a scalable and cost-effective strategy for lifestyle and exposome-related screening and may serve as a valuable preliminary step prior to targeted metabolomic analyses.

## Data Availability

The datasets generated and/or analyzed during the current study are available from the corresponding author on reasonable request.

## Declarations

### Source of biological material

Clinical data used in this study were obtained from participants recruited through the study approved by the Stockport Research Ethics Committee.
